# Temporal trends and regional disparities in prostate cancer mortality in the United States, 1999–2023: an analysis of the CDC WONDER database

**DOI:** 10.1186/s12889-025-25694-6

**Published:** 2025-12-18

**Authors:** Zixiong Chai, Sixiang Yan, Haolin Li, Xingyuan Dong, Songzhou Li, Yiming Fan, Zhiwu Dong, Zhiwei He, Jianbing Zhou, Pengkai Lei, Peng Gu

**Affiliations:** Department of Urology, The First Affiliated Hospital, Kunming Medical University, Kunming, Yunnan China

**Keywords:** Prostate cancer, Mortality trends, Racial disparities, Geographic variation, Rural-urban differences

## Abstract

**Background:**

Prostate cancer continues to be a major cause of cancer-related deaths among men in the United States. Despite declining mortality rates in recent decades, variations across race, region, urban-rural status, and age remain insufficiently characterized. Therefore, updated evidence is essential to inform effective prevention and control strategies.

**Methods:**

Prostate cancer deaths (ICD-10 code C61) among the U.S. men aged ≥ 45 years from 1999 to 2023 were analyzed using the CDC WONDER database. Age-adjusted mortality rate (AAMR) was calculated per 100,000 population and standardized to the 2000 U.S. standard population. Temporal trends were examined using joinpoint regression to estimate annual percent change (APC) and average APC. Subgroup analyses were conducted by region, race, urban-rural status, and age, with additional assessments at the state level.

**Results:**

Over the 25-year study period, 749,932 prostate cancer-related deaths were reported. Mortality initially declined but began to rise after 2013, reaching a peak of 33,860 deaths in 2023, reflecting a ‘decline followed by rebound’ pattern. Importantly, the increase in absolute deaths primarily reflects the effects of population aging. The AAMR decreased significantly overall (AAPC, −2.20%; *P* < .05), although the pace of decline slowed after 2013. This is likely influenced by changes in Prostate-Specific Antigen screening practices. Regionally, the South consistently exhibited the highest mortality, while the Northeast achieved the largest reduction and the West the smallest decline. By race, non-Hispanic (NH) Black men experienced the sharpest decrease yet continued to face the highest mortality, nearly twice that of NH White men. Mortality remained elevated in nonmetropolitan compared with metropolitan areas, and men aged 75 years and older continued to face the greatest risk.

**Conclusions:**

From 1999 to 2023, prostate cancer mortality among U.S. men aged 45 years and older showed a consistent decline. While the AAMR continued to decrease, the rate of decline slowed after 2013, likely due to changes in prostate-specific antigen (PSA) screening practices. In contrast, the observed rise in the absolute number of deaths mainly reflects demographic effects associated with population aging. The greatest burdens were observed among residents of the South, NH Black men, older adults, and rural populations. State-level AAMRs revealed a clear geographic gradient, with consistently higher rates in the South and lower rates in the Northeast. These findings highlight the need for future prevention and control strategies to prioritize high-risk groups through targeted interventions that promote health equity.

## Introduction

Prostate cancer is the most frequently diagnosed malignancy among men worldwide. Additionally, it remains one of the leading causes of cancer-related mortality in developed countries, posing a major public health challenge [1, 2]. Despite advances in prostate-specific antigen (PSA) screening, imaging technologies, and therapeutic strategies, the disease burden remains substantial. In the United States, prostate cancer remains one of the leading malignancies in both incidence and mortality among men. In 2025, it is projected to cause 313,780 new cases and 35,770 deaths, accounting for about 30% of all male cancer diagnoses. This highlights its significant public health burden and persistent racial disparities, as non-Hispanic (NH) Black men experience more than twice the mortality rate of NH White men[3].

Although epidemiologic studies of prostate cancer in the U.S. have advanced understanding of disease patterns, most nationwide analyses extend only through 2020 or earlier [4, 5]. Moreover, recent studies have adopted a multidimensional approach to simultaneously assess temporal trends and disparities across race and ethnicity, geographic regions, urban–rural residence, and age. Current evidence reveals disproportionately high mortality among NH Black men and a heavier disease burden in the Southern U.S[4–6]. However, many of these studies are constrained by limited scope or outdated data. Moreover, since the 2012 revision of the PSA screening recommendations by the U.S. Preventive Services Task Force (USPSTF)[7], relatively few investigations have assessed the long-term impact of this policy change. Consequently, it remains uncertain whether the recent decline in prostate cancer mortality has stabilized or begun to reverse [4, 8, 9]. Comprehensive nationwide analyses are urgently needed to evaluate prostate cancer mortality trends across temporal, demographic, and geographic dimensions. Such assessments are crucial for identifying high-risk populations and priority regions, thereby supporting evidence-based, targeted public health strategies and promoting equitable healthcare resource allocation.

To address these gaps, this study provides a comprehensive and current analysis of prostate cancer mortality among U.S. men aged 45 years and older from 1999 to 2023. Using the latest data from the Centers for Disease Control and Prevention Wide-ranging Online Data for Epidemiologic Research (CDC WONDER), we analyzed temporal trends, disparities across race, region, urban–rural classification, and age, as well as spatial heterogeneity at the state level. The findings aim to inform targeted public health interventions and support a more equitable distribution of healthcare resources.

## Method

### Data source

Data for this study were obtained from the U.S. Centers for Disease Control and Prevention Wide-ranging Online Data for Epidemiologic Research (CDC WONDER) database (https://wonder.cdc.gov/). This database compiles mortality records from death certificates across all U.S. counties and states, providing comprehensive national coverage. We extracted annual prostate cancer mortality data (ICD-10 code C61) for men aged 45 years and older from 1999 to 2023. Variables included the annual number of deaths, age-adjusted mortality rates (AAMRs), and demographic factors such as age group, race, urban–rural residence, census region, and state.

### Study population and subgroups

The study population included U.S. men aged 45 years and older, categorized into five age groups: 45–54, 55–64, 65–74, 75–84, and ≥ 85 years. Race and ethnicity were classified as NH White, NH Black, Hispanic, and NH Other. Urban–rural residence was determined using the 2013 U.S. Department of Agriculture Rural–Urban Continuum Codes (RUCCs) and categorized as metropolitan or non-metropolitan. Geographic regions followed U.S. Census Bureau definitions: Northeast, Midwest, South, and West. For spatial analyses, all 50 states and the District of Columbia were included.

### Definitions of measures

The annual number of deaths refers to the recorded count of prostate cancer-related deaths for each year. The AAMR was defined as the number of deaths per 100,000 population, standardized to the 2000 U.S. standard population. Percent change (PC) represents the percentage difference in mortality counts between 1999 and 2023. The annual PC (APC) quantifies the yearly rate of change in mortality within each time segment, whereas the average APC (AAPC) denotes the weighted average of APCs across the entire study period.

### Statistical analysis

Temporal trends in prostate cancer mortality from 1999 to 2023 were analyzed using the Joinpoint Regression Program (version 5.1.0.0, National Cancer Institute, USA). This method detects statistically significant changes in trends over time and estimates the APC for each segment. The AAPC was calculated as a weighted average of APCs across the entire study period to provide an overall trend estimate. Stratified analyses by age group, race, urban-rural status, census region, and state were conducted to evaluate disparities. Trends were considered statistically significant when the 95% confidence interval (CI) excluded zero (*P* <.05). Data visualization was performed using R software (version 4.5.1).

### Ethics statement

The CDC WONDER database provides publicly available, aggregate-level data without personal identifiers. Accordingly, institutional review board approval was not required for this study.

## Result

### Overall trends in prostate cancer mortality among U.S. Men aged 45 years and Older, 1999–2023

**Fig. 1:**
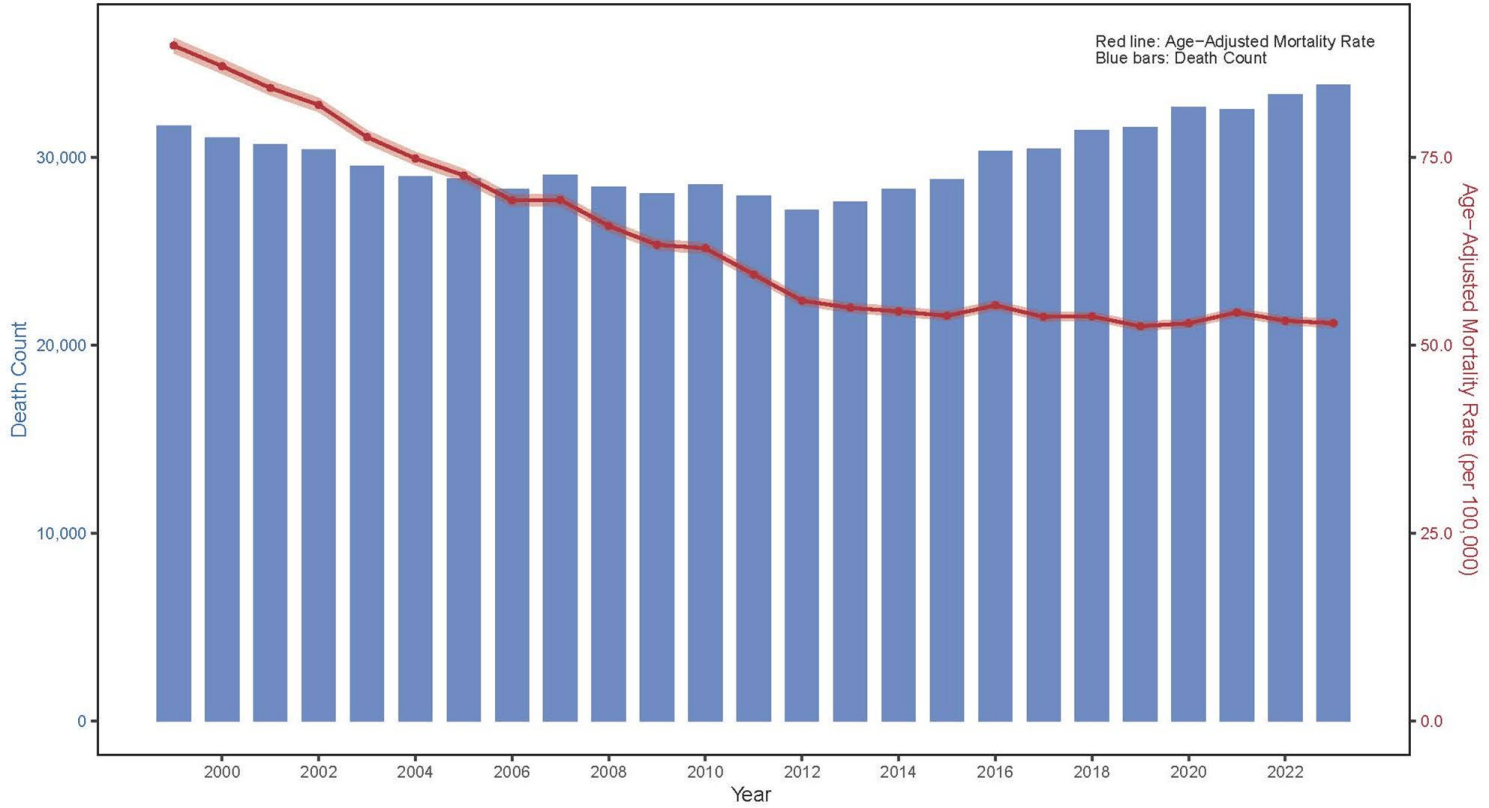
Temporal Trends in Prostate Cancer Deaths and Age-Adjusted Mortality Among U.S. Men Aged ≥ 45 Years, 1999–2023

Between 1999 and 2023, a total of 749,932 prostate cancer-related deaths were recorded among U.S. men aged 45 years and older (Table 1). Mortality exhibited a ‘decline-then-rebound’ pattern: deaths decreased from 31,691 in 1999 to 27,217 in 2012, representing a 14.12% reduction, and then gradually increased, peaking at 33,860 in 2023, a 6.84% rise from the 1999 baseline and the highest level observed during the 25-year period (Tables 2 and 3; Fig. 1).

**Table 1:** Prostate cancer deaths among U.S. Men Aged ≥ 45 Years, 1999–2023

Characteristic	Overall Deaths, 1999–2023, *n*		
Sex			
Male	749,932		

**Table 2:** Overall trends in prostate cancer mortality among U.S. Men aged ≥ 45 Years, 1999–2023, with stratified analyses by census Region, Race, Urban–Rural Residence, and age

Characteristic	Deaths			AAMR (per 100,000)		
	1999, *n*	2023, *n*	PC (%)	1999 (95% CI)	2023 (95% CI)	AAPC, % (95% CI)
Sex						
Male	31,691	33,860	6.84	89.87 (88.86 to 90.88)	52.93 (52.36 to 53.51)	−2.20 (−2.36 to −2.05)^a^
Census Region						
Northeast	6421	5422	−15.56	87.69 (85.51 to 89.87)	46.95 (45.68 to 48.22)	−2.62 (−2.86 to −2.39) ^a^
Midwest	7670	7304	−4.77	91.91 (89.82 to 94.00)	54.80 (53.51 to 56.08)	−2.17 (−2.33 to −2.00) ^a^
South	11,562	12,761	10.37	93.98 (92.22 to 95.73)	52.33 (51.41 to 53.26)	−2.35 (−2.52 to −2.18) ^a^
West	6038	8373	38.67	82.77 (80.65 to 84.90)	56.91 (55.67 to 58.15)	−1.61 (−1.84 to −1.37) ^a^
Race						
Hispanic	1046	2503	139.29	65.86 (61.62 to 70.10)	42.52 (40.77 to 44.26)	−2.06 (−2.41 to −1.71) ^a^
NH Black	5308	5630	6.07	199.91 (194.35 to 205.47)	102.12 (99.27 to 104.96)	−2.91 (−3.22 to −2.59) ^a^
NH White	24,910	24,590	−1.28	82.98 (81.93 to 84.03)	51.17 (50.52 to 51.82)	−1.99 (−2.16 to −1.82)^a^
NH Other	362	1050	190.06	41.57 (37.10 to 46.05)	24.25 (22.77 to 25.74)	−2.00 (−2.29 to −1.72) ^a^
Urbanization						
Metropolitan	25,104	27,939	11.29	88.44 (87.33 to 89.56)	52.39 (51.75 to 53.02) ^b^	−2.48 (−2.67 to −2.28) ^a b^
Nonmetropolitan	6587	5921	−10.11	95.66 (93.31 to 98.00)	55.68 (54.23 to 57.12) ^b^	−2.54 (−2.79 to −2.29) ^a b^
Age group ^c^, y						
45–54	374	329	−12.03	2.08 (2.08 to 2.08)	1.63 (1.63 to 1.63)	−0.73 (−1.13 to −0.33) ^a^
55–64	1946	2718	39.67	17.07 (17.07 to 17.07)	13.26 (13.26 to 13.26)	−0.93 (−1.37 to −0.49) ^a^
65–74	7316	8366	14.35	88.21 (88.21 to 88.21)	51.21 (51.21 to 51.21)	−2.11 (−2.41 to −1.80) ^a^
75–84	13,435	11,996	−10.71	279.10 (279.10 to 279.10)	146.96 (146.96 to 146.96)	−2.59 (−2.75 to −2.44) ^a^
≥ 85	8620	10,451	21.24	722.36 (722.36 to 722.36)	457.58 (457.58 to 457.58)	−2.08 (−2.38 to −1.79) ^a^

*Abbreviation*: *AAMR* Age-Adjusted Mortality Rate (per 100,000 persons), *AAPC* Annual Average Percent Change, *CI* Confidence Interval, *PC* Percentage Change, *NH* Non-Hispanic

^a^ It was statistically significant at *P* <.05

^b^ For urbanization levels, AAMR values for 2023 were derived from 2020 data; AAPC was calculated using data from 1999–2020

^c^ For age subgroups, both AAMR and AAPC were calculated using crude mortality rates

**Table 3:** Annual prostate cancer deaths in the united States, 1999–2023

Year	Deaths, *n*	AAMR (per 100,000)	Year	Deaths, *n*	AAMR (per 100,000)
1999	31,691	89.87	2012	27,217	55.92
2000	31,052	87.12	2013	27,655	54.98
2001	30,687	84.22	2014	28,320	54.49
2002	30,412	81.97	2015	28,826	53.91
2003	29,526	77.69	2016	30,347	55.30
2004	28,974	74.84	2017	30,475	53.78
2005	28,876	72.60	2018	31,463	53.83
2006	28,325	69.26	2019	31,607	52.53
2007	29,069	69.33	2020	32,672	52.92
2008	28,446	65.87	2021	32,541	54.37
2009	28,069	63.37	2022	33,338	53.25
2010	28,537	62.91	2023	33,860	52.93
2011	27,947	59.42			

*Abbreviation*: *AAMR* Age-adjusted mortality rate (per 100,000 persons)

**Fig. 2:**
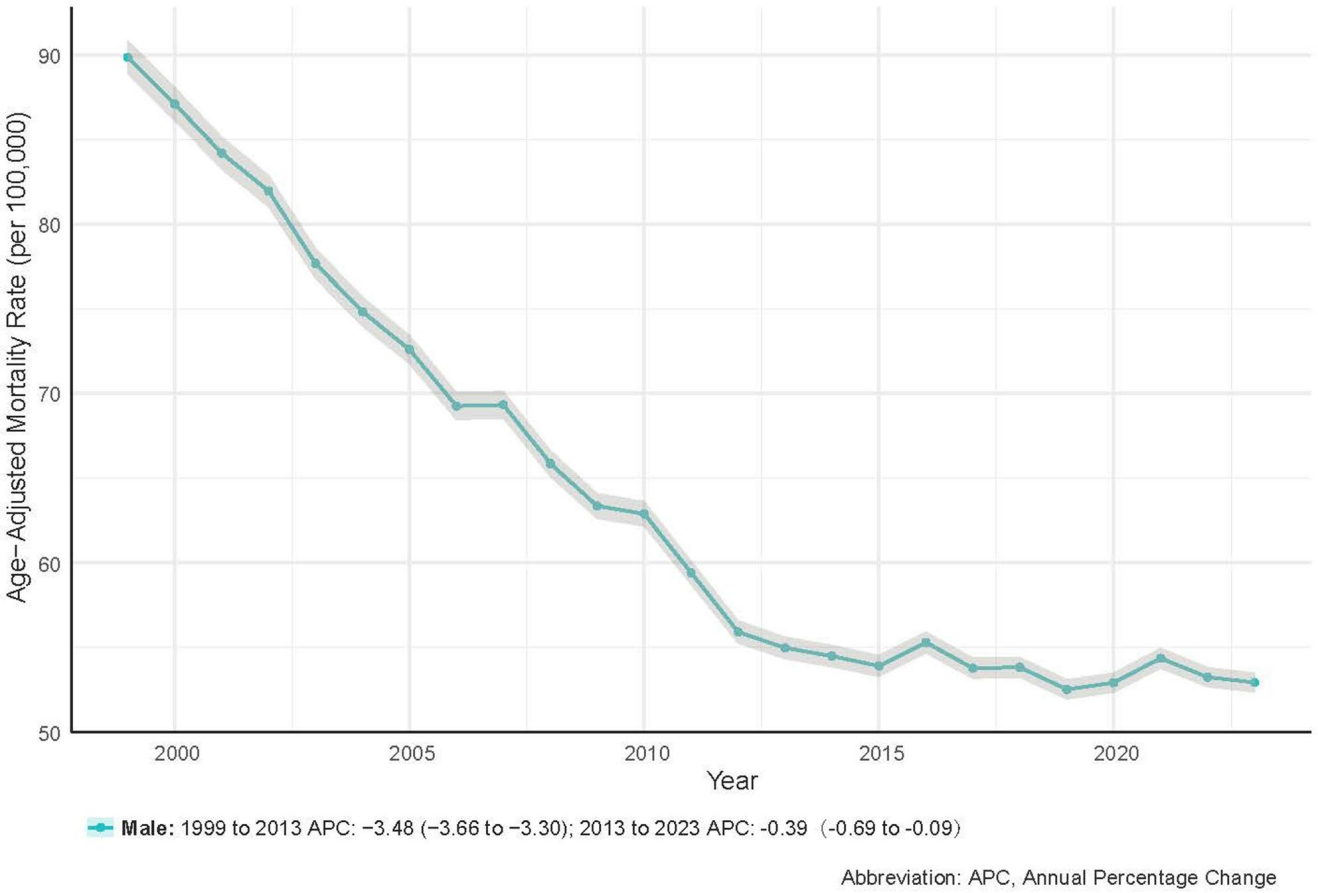
Joinpoint Regression Analysis of Prostate Cancer Age-Adjusted Mortality Rates, 1999–2023

As shown in Tables 2 and 3; Fig. 1, the AAMR declined steadily from 89.87 per 100,000 in 1999 to 52.93 per 100,000 in 2023, with an AAPC of −2.20% (*P* <.05). Joinpoint regression indicated a steeper decline between 1999 and 2013 (APC = −3.48%, *P* >.05), followed by a pronounced slowdown after 2013 (APC = −0.39%, *P* >.05) (Fig. 2).

### Stratified mortality trends and disparities

#### Census region

According to Table 4, the South carried the greatest mortality burden, accounting for 275,857 deaths (36.78%), whereas the Northeast had the fewest deaths at 138,860 (18.52%). Between 1999 and 2023, mortality counts increased most in the West (+ 38.67%), while declines were observed in the Northeast (−15.56%) and Midwest (−4.77%).

**Table 4:** Prostate cancer mortality burden by U.S. Census Region, 1999–2023

Census Region	Overall Deaths, 1999–2023, *n*
Northeast	138,860
Midwest	169,829
South	275,857
West	165,386

**Fig. 3:**
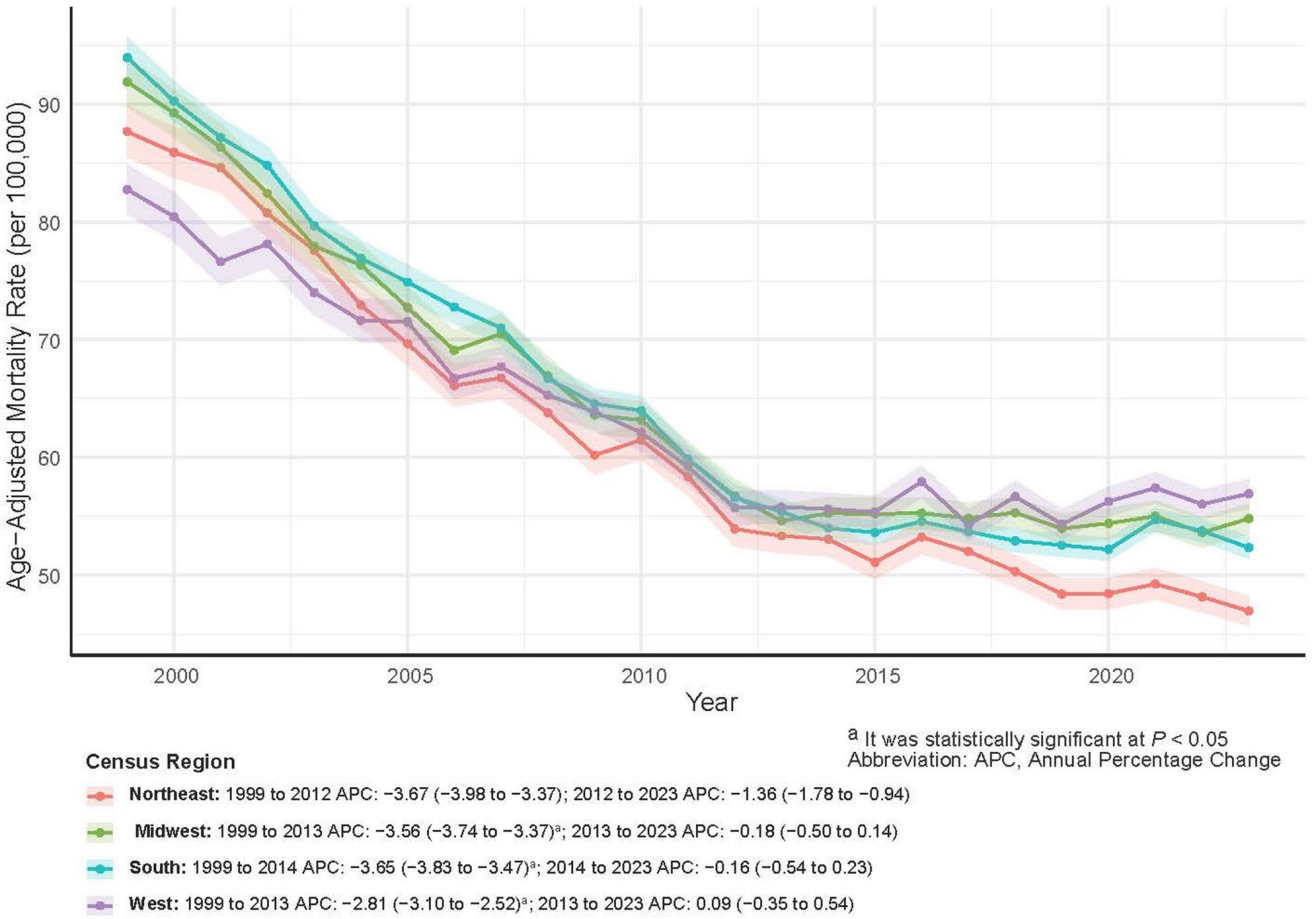
Trends in Prostate Cancer Age-Adjusted Mortality Rates by U.S. Census Region, 1999–2023

AAMR decreased significantly across all regions, although the magnitude of decline varied (Table 2; Fig. 3). The Northeast experienced the largest reduction, from 87.69 to 46.95 per 100,000 (AAPC = −2.62%), whereas the West showed the smallest decline (AAPC = −1.61%). Both the South and Midwest followed a pattern of steep early declines followed by slower decreases over time.

#### Race

**Fig. 4:**
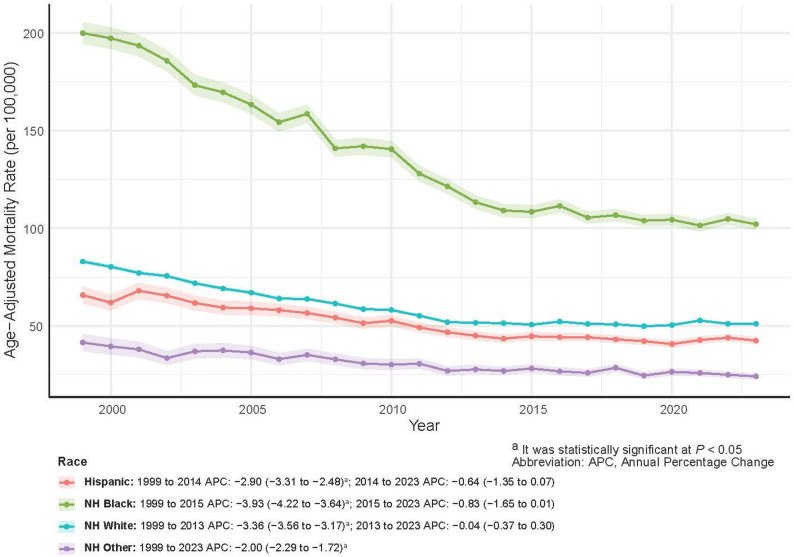
Prostate Cancer AAMRs and APC by Race, U.S. Men, 1999–2023

As shown in Table 5, NH White men accounted for the largest number of deaths (567,411; 75.66%), whereas NH Other men had the fewest (15,453; 2.06%). NH Black men consistently experienced the highest AAMR and the steepest decline over time, yet their rates remained nearly twice those of NH White men. Hispanic and NH Other groups exhibited relatively low AAMRs with steady downward trends (AAPC = −2.06% and − 2.00%, respectively). From 1999 to 2023, the NH Other group experienced the largest relative increase in deaths (+ 190.06%, from 362 to 1,050), while NH White men saw a slight decline (−1.28%, from 24,910 to 24,590 deaths) (Table 2; Fig. 4). Although racial disparities narrowed over time, they persisted throughout the study period.

**Table 5:** Prostate cancer mortality burden by race in the united States, 1999–2023

Race	Overall Deaths, 1999–2023, *n*
Hispanic	41,415
NH Black	124,065
NH White	567,411
NH Other	15,453

*Abbreviation*: *NH* Non-Hispanic

#### Urban-Rural differences

**Fig. 5:**
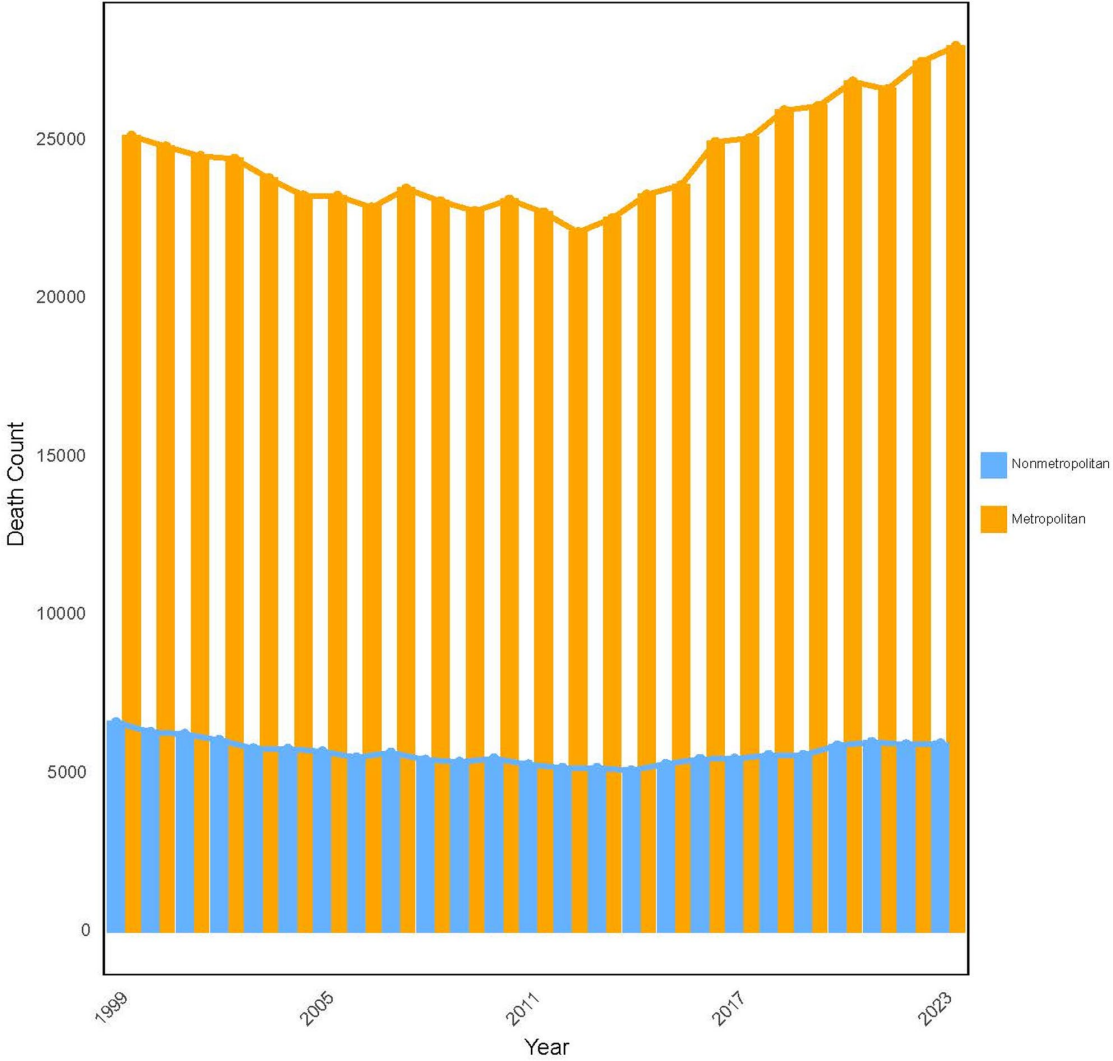
Annual Prostate Cancer Deaths in Metropolitan vs. Nonmetropolitan U.S. Areas, 1999–2023

**Fig. 6:**
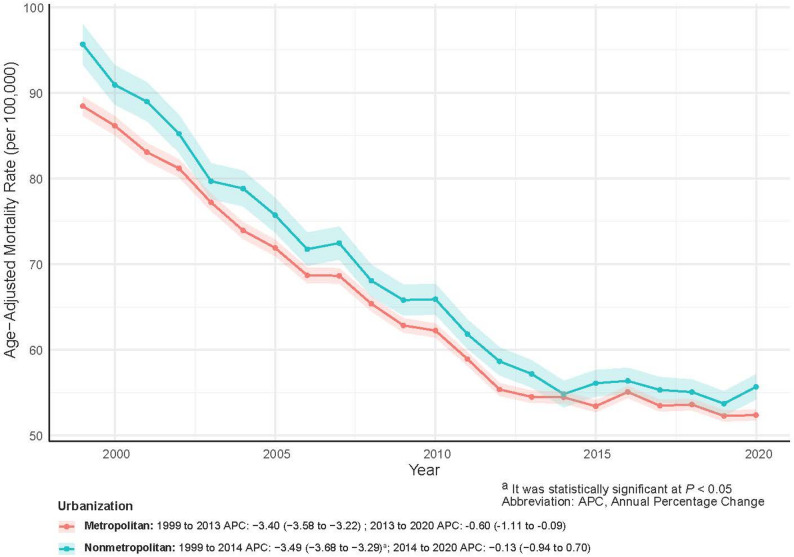
Prostate Cancer AAMRs and APC in Metropolitan vs. Nonmetropolitan U.S. Areas, 1999–2023

Metropolitan areas accounted for the majority of prostate cancer deaths (608,806; 81.18%), reflecting an 11.29% increase, whereas nonmetropolitan areas recorded 141,126 deaths (18.82%), corresponding to a 10.11% decline (Tables 2 and 6; Fig. 5). From 1999 to 2023, both metropolitan and nonmetropolitan areas experienced significant declines in AAMR (AAPC = −2.54% and − 2.48%, respectively). However, AAMR in nonmetropolitan areas remained consistently higher, and the urban-rural mortality gap did not narrow over time (Table 2; Fig. 6).

**Table 6:** Burden of prostate cancer deaths by Urban-Rural residence in the united States, 1999–2023

Urbanization	Overall Deaths, 1999–2023, *n*
Nonmetropolitan	141,126
Metropolitan	608,806

#### Age groups

**Fig. 7:**
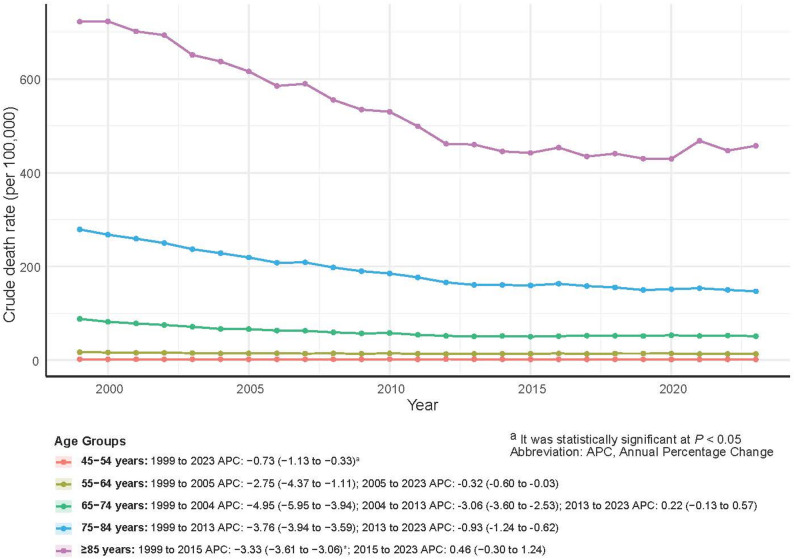
Prostate Cancer Crude Mortality and APC by Age Group, U.S. Men, 1999–2023

All age groups experienced declines in mortality rates, although the levels and magnitudes of decline varied. Mortality was concentrated among older adults and increased with age. Men aged ≥ 85 years consistently had the highest rates, reaching 457.58 per 100,000 in 2023. Despite a significant decline in AAMR (AAPC = −2.08%, *P* <.05), deaths in this age group increased by 21.24%. The 65–74 and 75–84 age groups contributed the largest absolute numbers of deaths (8,366 and 11,996, respectively). In contrast, men aged 45–54 years had the lowest mortality rates, minimal decline, and a limited contribution to the overall burden (Table 2; Fig. 7).

#### State-level patterns

**Fig. 8:**
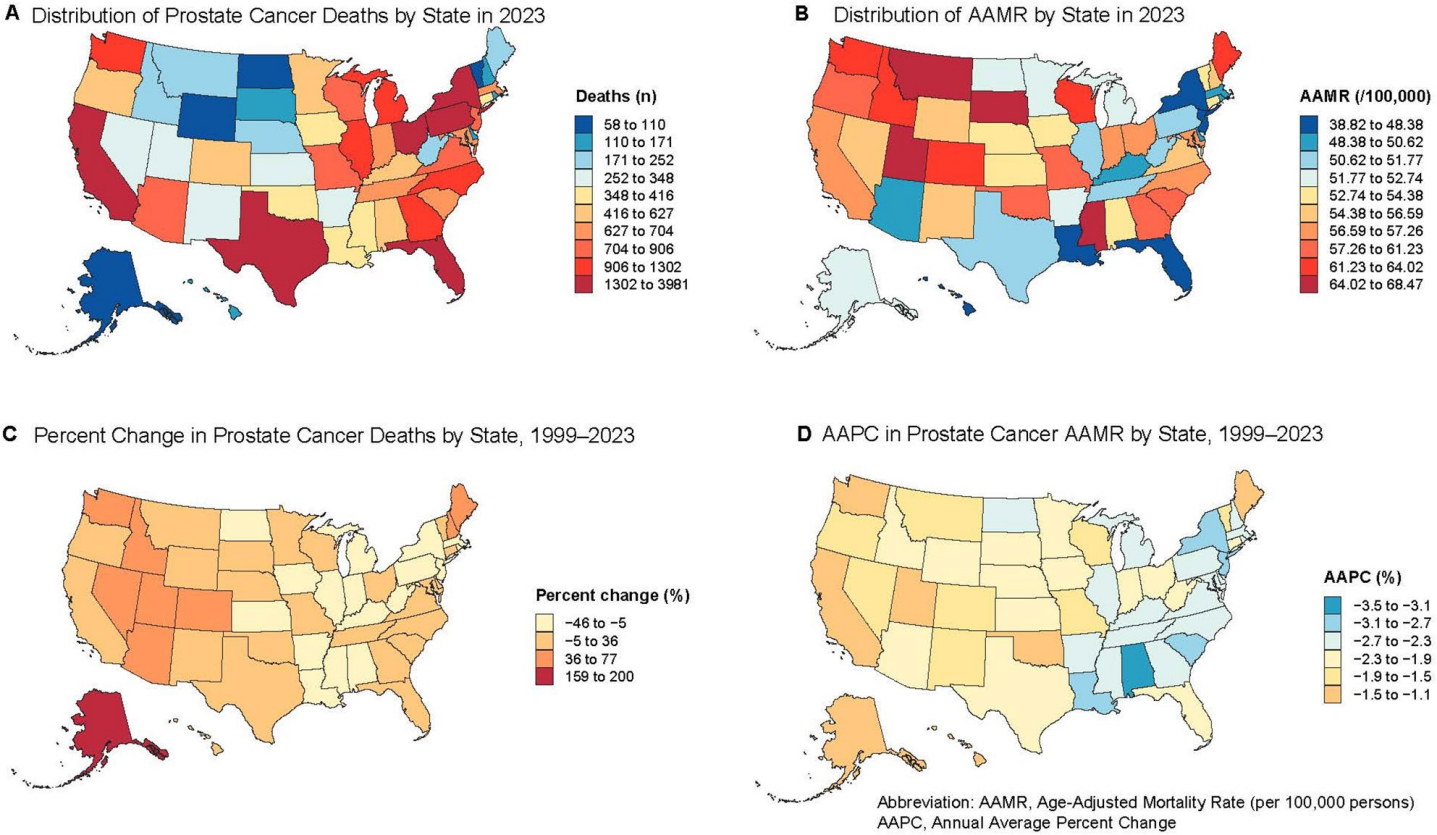
Geographic Distribution and Trends in Prostate Cancer Mortality Across U.S. States, 1999–2023. Note: Panel **A**: Distribution of Prostate Cancer Deaths by State in 2023; Panel **B**: Distribution of Age-Adjusted Mortality Rates (AAMRs) by State in 2023; Panel **C**: Percent Change in Prostate Cancer Deaths by State, 1999–2023; Panel **D**: Average Annual Percent Change (AAPC) in Prostate Cancer AAMRs by State, 1999–2023

The mortality burden showed a clear dependence on population size, with more densely populated states bearing a heavier burden. California (81,851 deaths), Florida (56,648), Texas (45,039), New York (44,761), Pennsylvania (36,078), and Ohio (30,197) together accounted for approximately 40% of all deaths. Other high-burden states included Illinois (30,793), Michigan (24,567), North Carolina (23,159), Georgia (20,627), and New Jersey (20,356), primarily concentrated in the Northeast, South, and Midwest. States with smaller populations, such as Wyoming (1,317), Vermont (1,765), North Dakota (1,966), the District of Columbia (2,004), and Delaware (2,370), each recorded fewer than 3,000 deaths (Table 7; Fig. 8A). Mortality counts rose sharply in large states, including California and Texas, whereas declines of approximately 18–19% were observed in New York and Pennsylvania. Even smaller states, such as Wyoming, experienced increases in mortality (Table 7; Fig. 8C).

**Table 7:** Prostate cancer mortality burden by state in the united states, 1999–2023

State	Overall Deaths, 1999–2023, *n*	State	Overall Deaths, 1999–2023, *n*
Alabama	13,197	Montana	3189
Alaska	1018	Nebraska	4662
Arizona	15,516	Nevada	6224
Arkansas	7813	New Hampshire	3375
California	81,851	New Jersey	20,356
Colorado	10,945	New Mexico	5173
Connecticut	9235	New York	44,761
Delaware	2370	North Carolina	23,159
District of Columbia	2004	North Dakota	1966
Florida	56,648	Ohio	30,197
Georgia	20,627	Oklahoma	9345
Hawaii	2958	Oregon	11,090
Idaho	4290	Pennsylvania	36,078
Illinois	30,793	Rhode Island	2788
Indiana	15,695	South Carolina	12,716
Iowa	8802	South Dakota	2364
Kansas	6772	Tennessee	15,539
Kentucky	9989	Texas	45,039
Louisiana	11,168	Utah	5232
Maine	4017	Vermont	1765
Maryland	13,812	Virginia	18,776
Massachusetts	16,485	Washington	16,583
Michigan	24,567	West Virginia	4928
Minnesota	13,729	Wisconsin	15,988
Mississippi	8727	Wyoming	1317
Missouri	14,294		

State-level AAMRs declined overall, yet substantial geographic disparities persisted (Tables 7 and 8). In 2023, Southern states such as Mississippi and Georgia, along with the District of Columbia, had the highest rates (> 60 per 100,000), exceeding the national average, whereas states primarily in the Northeast and on the West Coast (e.g., Hawaii, New Jersey, and New York) had rates below 45 per 100,000, illustrating a clear ‘higher in the South, lower in the Northeast’ pattern (Table 8; Fig. 8B). AAPC analysis indicated the largest declines (≤ −3.0%) in several Southern and Northeastern states (e.g., DC, Alabama, New York), while some Western states (e.g., Washington, Maine) exhibited minimal reductions or stable rates (Table 8; Fig. 8D).

**Table 8:** Prostate cancer mortality and trends by state among U.S. Men Aged ≥ 45 Years, 1999–2023

State	Deaths			AAMR (per 100,000)			
	1999, *n*	2023, *n*	PC (%)	1999 (95% CI)	2023 (95% CI)		AAPC, % (95% CI)
Alabama	613	535	−12.72	112.35 (103.18 to 121.51)		54.38 (49.64 to 59.11)	−3.13 (−3.66 to −2.60)^a^
Alaska	20	59	195.00	59.30 (34.54 to 94.94)		51.82 (38.21 to 68.70)	−1.32 (−2.41 to −0.22) ^a^
Arizona	498	804	61.45	69.84 (63.54 to 76.14)		48.38 (45.01 to 51.75)	−2.02 (−2.51 to −1.52) ^a^
Arkansas	378	312	−17.46	101.06 (90.71 to 111.41)		51.77 (45.91 to 57.63)	−2.39 (−3.10 to −1.68) ^a^
California	3075	3980	29.43	81.62 (78.69 to 84.56)		56.80 (55.01 to 58.59)	−1.45 (−1.71 to −1.20) ^a^
Colorado	361	603	67.04	85.61 (76.54 to 94.68)		61.29 (56.22 to 66.36)	−1.50 (−2.13 to −0.88) ^a^
Connecticut	404	395	−2.23	82.61 (74.46 to 90.76)		52.90 (47.61 to 58.19)	−1.98 (−2.60 to −1.35) ^a^
Delaware	93	112	20.43	94.28 (75.31 to 116.58)		50.32 (40.75 to 59.89)	−2.44 (−3.32 to −1.55) ^a^
District of Columbia	113	64	−43.36	166.09 (134.93 to 197.25)		65.34 (50.09 to 83.76)	−3.23 (−3.92 to −2.55) ^a^
Florida	2243	2653	18.28	74.19 (71.08 to 77.30)		45.76 (44.01 to 47.51)	−1.96 (−2.26 to −1.65) ^a^
Georgia	813	1081	32.96	111.42 (103.43 to 119.41)		61.23 (57.43 to 65.02)	−2.40 (−2.80 to −2.00) ^a^
Hawaii	116	139	19.83	64.68 (52.84 to 76.52)		38.83 (32.33 to 45.33)	−1.44 (−2.60 to −0.27) ^a^
Idaho	159	235	47.80	96.02 (80.94 to 111.09)		64.02 (55.62 to 72.42)	−1.94 (−2.35 to −1.54) ^a^
Illinois	1419	1223	−13.81	94.60 (89.59 to 99.61)		51.36 (48.42 to 54.30)	−2.50 (−2.86 to −2.14) ^a^
Indiana	746	695	−6.84	100.87 (93.47 to 108.27)		57.26 (52.88 to 61.64)	−2.08 (−2.57 to −1.58) ^a^
Iowa	416	348	−16.35	90.61 (81.86 to 99.36)		52.74 (47.11 to 58.38)	−2.17 (−2.79 to −1.54) ^a^
Kansas	313	293	−6.39	83.80 (74.46 to 93.13)		52.81 (46.66 to 58.96)	−2.23 (−2.94 to −1.51) ^a^
Kentucky	461	416	−9.76	95.13 (86.18 to 104.09)		49.46 (44.53 to 54.39)	−2.51 (−3.19 to −1.83) ^a^
Louisiana	550	402	−26.91	110.37 (100.87 to 119.88)		47.09 (42.33 to 51.84)	−3.09 (−3.68 to −2.49) ^a^
Maine	148	210	41.89	79.68 (66.59 to 92.77)		63.03 (54.28 to 71.77)	−1.13 (−2.45 to 0.22)
Maryland	580	646	11.38	99.38 (91.00 to 107.75)		56.78 (52.31 to 61.25)	−2.34 (−2.76 to −1.93) ^a^
Massachusetts	801	664	−17.10	95.17 (88.48 to 101.85)		50.23 (46.34 to 54.12)	−2.50 (−2.82 to −2.18) ^a^
Michigan	1137	1047	−7.92	93.41 (87.84 to 98.97)		51.91 (48.68 to 55.15)	−2.34 (−2.82 to −1.85) ^a^
Minnesota	566	581	2.65	89.57 (82.13 to 97.00)		52.03 (47.73 to 56.33)	−2.14 (−4.40 to 0.16)
Mississippi	426	376	−11.74	128.70 (116.26 to 141.15)		68.34 (61.19 to 75.49)	−2.49 (−3.21 to −1.77) ^a^
Missouri	662	704	6.34	87.82 (81.02 to 94.62)		57.92 (53.56 to 62.29)	−1.59 (−2.70 to −0.46) ^a^
Montana	129	171	32.56	99.75 (82.32 to 117.18)		68.46 (57.94 to 78.98)	−1.79 (−2.44 to −1.14) ^a^
Nebraska	188	196	4.26	77.25 (66.13 to 88.37)		53.10 (45.54 to 60.67)	−1.91 (−2.37 to −1.45) ^a^
Nevada	196	343	75.00	84.60 (71.79 to 97.41)		55.26 (49.25 to 61.26)	−1.72 (−2.47 to −0.96) ^a^
New Hampshire	118	168	42.37	82.17 (66.95 to 97.39)		54.71 (46.19 to 63.22)	−2.34 (−2.94 to −1.74) ^a^
New Jersey	965	731	−24.25	87.11 (81.50 to 92.71)		41.09 (38.06 to 44.12)	−2.99 (−3.52 to −2.46) ^a^
New Mexico	197	252	27.92	91.44 (78.39 to 104.49)		55.78 (48.76 to 62.81)	−1.88 (−2.51 to −1.25) ^a^
New York	2055	1673	−18.59	84.29 (80.58 to 87.99)		42.52 (40.46 to 44.59)	−3.00 (−3.21 to −2.79) ^a^
North Carolina	908	1108	22.03	100.74 (93.89 to 107.59)		56.68 (53.23 to 60.13)	−2.58 (−2.99 to −2.17) ^a^
North Dakota	88	77	−12.50	82.39 (66.08 to 101.50)		52.48 (41.28 to 65.78)	−2.68 (−3.38 to −1.97) ^a^
Ohio	1341	1302	−2.91	91.11 (86.10 to 96.12)		56.88 (53.71 to 60.05)	−2.16 (−2.60 to −1.71) ^a^
Oklahoma	392	415	5.87	86.58 (77.85 to 95.31)		57.33 (51.68 to 62.98)	−1.49 (−1.96 to −1.02) ^a^
Oregon	442	526	19.00	93.12 (84.35 to 101.88)		59.60 (54.37 to 64.83)	−1.74 (−2.25 to −1.22) ^a^
Pennsylvania	1718	1391	−19.03	90.95 (86.57 to 95.34)		50.62 (47.91 to 53.33)	−2.31 (−2.61 to −2.01) ^a^
Rhode Island	133	112	−15.79	87.88 (72.72 to 103.04)		49.85 (40.44 to 59.27)	−2.33 (−2.95 to −1.71) ^a^
South Carolina	533	627	17.64	117.99 (107.44 to 128.53)		59.30 (54.47 to 64.13)	−2.90 (−3.33 to −2.46) ^a^
South Dakota	114	110	−3.51	95.90 (78.22 to 113.58)		64.09 (51.83 to 76.36)	−2.08 (−3.22 to −0.93) ^a^
Tennessee	660	673	1.97	99.80 (91.95 to 107.65)		51.15 (47.14 to 55.16)	−2.60 (−3.10 to −2.10) ^a^
Texas	1774	2253	27.00	87.13 (82.95 to 91.30)		51.11 (48.93 to 53.29)	−2.16 (−2.42 to −1.89) ^a^
Utah	190	283	48.95	93.64 (80.07 to 107.22)		64.71 (56.98 to 72.45)	−1.50 (−2.40 to −0.58) ^a^
Vermont	79	78	−1.27	101.46 (79.94 to 127.00)		53.81 (42.17 to 67.65)	−1.72 (−2.56 to −0.88) ^a^
Virginia	784	875	11.61	103.29 (95.74 to 110.84)		54.82 (51.09 to 58.54)	−2.39 (−2.89 to −1.89) ^a^
Washington	599	906	51.25	84.25 (77.41 to 91.08)		63.44 (59.21 to 67.68)	−1.38 (−2.75 to 0.01)
West Virginia	241	213	−11.62	88.41 (76.96 to 99.86)		51.56 (44.47 to 58.66)	−2.05 (−3.14 to −0.95) ^a^
Wisconsin	680	728	7.06	93.41 (86.31 to 100.51)		61.26 (56.69 to 65.83)	−1.79 (−2.19 to −1.39) ^a^
Wyoming	56	72	28.57	92.29 (69.13 to 120.72)		56.59 (43.94 to 71.74)	−2.22 (−4.22 to −0.19) ^a^

*Abbreviation*: *AAMR* Age-Adjusted Mortality Rate (per 100,000 persons), *AAPC* Annual Average Percent Change, *CI* Confidence Interval, *PC* Percentage Change

^a^ It was statistically significant at *P* <.05

### Summary

Between 1999 and 2023, prostate cancer mortality among U.S. men aged 45 years and older declined overall, though the rate of decline slowed substantially after 2013. Marked disparities persisted by region, race, urban-rural residence, and age, with the highest mortality observed among NH Black men, older adults, residents of the South, and those in nonmetropolitan areas. These findings underscore persistent challenges that must be addressed to enhance prostate cancer prevention, control, and equity in outcomes.

## Discussion

This study systematically examined prostate cancer mortality among U.S. men aged 45 years and older from 1999 to 2023 using the CDC WONDER database. Over the 25-year period, the AAMR declined steadily from 89.87 to 52.93 per 100,000 (AAPC = − 2.20%), reflecting sustained progress in reducing prostate cancer mortality. However, substantial disparities persisted across race, geographic region, and urban–rural residence. Notably, since 2013, mortality counts have increased and the pace of decline in AAMR has slowed, underscoring the complexity of the prostate cancer burden, ongoing health inequities, and emerging public health challenges.

The continued decline in AAMRs indicates that, after accounting for population age structure, prostate cancer–related mortality has decreased, reflecting the effectiveness of public health strategies [3]. This trend likely stems from the widespread adoption of PSA screening, advancements in diagnostic and therapeutic technologies, and the development of novel treatment options [10–12]. However, despite the overall reduction in risk, the absolute number of deaths has increased, reflecting population aging and resulting in a ‘decline followed by rebound’ pattern. This trend became more apparent following the 2012 recommendation by the U.S. Preventive Services Task Force (USPSTF) against routine PSA screening, which led to a subsequent decline in screening frequency [13, 14].,¹⁵ This decline in screening coincided with a marked slowdown in the decrease of AAMRs after 2013, with the APC shifting from − 3.48% to − 0.39%. Evidence indicates that following the 2012 USPSTF recommendation against routine PSA screening, the incidence of advanced and newly metastatic prostate cancer has risen annually, suggesting that reduced early detection may have contributed to the slower decline in AAMRs [15–20]. Multiple studies have confirmed that this policy change was linked to reduced PSA screening and detection rates, accompanied by increases in late-stage disease incidence and mortality [10, 12, 15, 21]. Importantly, the USPSTF now recommends individualized decision-making for prostate cancer screening, emphasizing shared discussions between patients and healthcare providers about the potential benefits and harms [22, 23]. However, men lacking access to primary care may encounter barriers to such discussions, reducing opportunities for informed decision-making and early detection [21]. Future research should more comprehensively examine healthcare access, as disparities in availability may affect screening participation, early diagnosis, and treatment outcomes. This highlights the need to address preventive healthcare services alongside post-diagnosis management to reduce inequities in prostate cancer outcomes. Our findings provide updated nationwide evidence that, although AAMRs continue to decline modestly, the absolute mortality burden is increasing, underscoring the need for public health strategies that address both risk reduction and the growing impact of deaths in an aging population.

Furthermore, the results highlight the multidimensional nature of health inequities. Racial disparities remain pronounced: NH Black men consistently exhibited the highest AAMRs, nearly twice those of NH White men. Although mortality among this group declined substantially, the disparity persisted. These inequities likely reflect a combination of genetic susceptibility, limited healthcare access, socioeconomic disadvantage, and differences in treatment adherence [24, 25]. Public health interventions focused on high-risk populations, particularly NH Black men, are therefore essential [26]. The persistent Black–White survival gap remains a topic of ongoing discussion. Evidence shows that NH Black men are more likely to develop aggressive prostate cancer and experience limited access to high-quality screening, treatment, and follow-up care. Several studies suggest that these disparities are largely attributable to socioeconomic inequalities and barriers to healthcare access [6, 27, 28]. Meanwhile, genetic studies have implicated HOXB13 variants and the 8q24 locus in prostate cancer progression, which may partially explain observed racial differences [29–33]. Differentiating the relative contributions of social determinants and biological factors remains a major challenge for future research. Integrating both dimensions will be essential to develop targeted interventions that effectively reduce prostate cancer disparities [34, 35]. 

Geographic and urban–rural disparities also shaped the spatial distribution of prostate cancer mortality. AAMRs were highest in the South, where the rate of decline was comparatively modest. This “high-burden, slow-improvement” pattern is strongly associated with higher poverty levels, lower insurance coverage, limited healthcare infrastructure, and a greater proportion of NH Black residents [36, 37]. Previous studies have likewise reported elevated mortality in the South and Midwest, attributing these patterns to socioeconomic disadvantage, limited healthcare access, and the concentration of high-risk populations [38]. In contrast, the Northeast experienced the steepest reductions, reflecting more effective screening and treatment programs [39]. State-level distribution demonstrated a ‘higher in the South, lower in the North’ pattern, with Southern states consistently at greater risk, whereas Northeastern states generally represented low-risk areas. These disparities largely stem from regional variations in health insurance coverage, public health funding allocation, and the development of cancer prevention and control infrastructures [38, 40]. Additionally, AAMRs in nonmetropolitan areas consistently exceeded those in metropolitan areas, with the decline plateauing after 2014, underscoring persistent urban-rural disparities. Prior studies suggest that rural residents have lower PSA screening rates and poorer treatment adherence, compounded by barriers such as transportation challenges, limited healthcare infrastructure, insufficient access to advanced medical technologies, and shortages of oncology specialists [41, 42]. Expanding access to screening and treatment in the South and rural areas will be essential to reducing these disparities.

Age-stratified analyses demonstrated that prostate cancer mortality was heavily concentrated among older adults. Men aged ≥ 75 years experienced substantially higher mortality than those aged 55–64, and rates among men aged ≥ 85 years have recently increased, reflecting the impact of aging-related disease processes[43]. Clinical decision-making for older patients is particularly complex, requiring careful consideration of oncologic benefits, comorbidities, functional status, and life expectancy [44, 45]. Striking a balance between avoiding overtreatment and ensuring appropriate screening and treatment for high-risk older adults remains a critical public health challenge [46].

This study has important public health implications. Although overall prostate cancer mortality has declined, the slowing pace of decline and the recent rebound in deaths underscore the growing impact of population aging. High-risk groups, including NH Black men, residents of the South, and rural populations, continue to bear disproportionate burdens. Future strategies should prioritize these populations by optimizing screening, strengthening early detection, and ensuring equitable access to effective treatment, with the goal of reducing mortality and narrowing disparities [14]. 

Several limitations should be acknowledged. The CDC WONDER database, which relies on death certificate data, lacks clinical details such as tumor stage, Gleason score, and treatment information, limiting the ability to explore underlying determinants of mortality. Additionally, AAMRs, which are standardized to a reference population, may not fully reflect demographic changes within specific subgroups. Future research should utilize linked datasets, such as the Surveillance, Epidemiology, and End Results–Medicare (SEER–Medicare) database and the National Cancer Database, to assess how socioeconomic status, healthcare access, and geographic factors contribute to disparities in prostate cancer mortality. Moreover, integrating environmental exposures (e.g., neighborhood deprivation, pollution levels) and molecular characteristics (e.g., tumor genomics, PSA kinetics) may help identify high-risk subgroups and clarify the causal pathways underlying racial and regional disparities. Such multidimensional analyses could inform targeted interventions to reduce inequities and improve early detection and treatment outcomes [6, 47]. 

## Conclusion

From 1999 to 2023, the AAMR for prostate cancer among U.S. men aged 45 years and older declined steadily; however, the rate of decline slowed after 2013, accompanied by a rebound in deaths that poses new challenges for disease control. Substantial disparities persisted across race, geography, urban–rural status, and age. Future prevention and control strategies should prioritize high-risk populations and regions. This approach should reinforce evidence-based screening consistent with current USPSTF recommendations while addressing barriers to both primary and specialty care to ensure timely diagnosis and appropriate management, including active surveillance when indicated. Additionally, efforts should promote equitable resource allocation and implement targeted interventions to improve prostate cancer prevention and control outcomes.

## Data Availability

The data used in this study were obtained from the publicly available CDC WONDER database ([https://wonder.cdc.gov/](https:/wonder.cdc.gov)). This resource provides open access to nationwide mortality data, including prostate cancer–related deaths (ICD-10 code C61) among U.S. men aged 45 years and older from 1999 to 2023. As the database contains only aggregate, de-identified information, no individual privacy is involved. All data analyzed in this study are publicly accessible through the CDC WONDER platform; therefore, no additional data sharing is required.

## Abbreviations

AAPC: Average Annual Percent Change
AAMR: Age-Adjusted Mortality Rate
APC: Annual Percent Change
PC: Percent change
CDC WONDER: Centers for Disease Control and Prevention - Wide-ranging Online Data for Epidemiologic Research
CI: Confidence Interval
ICD-10: International Classification of Diseases, Tenth Revision
NH: Non-Hispanic
PSA: Prostate-Specific Antigen
RUCCs: Rural-Urban Continuum Codes
SEER-Medicare: Surveillance, Epidemiology, and End Results–Medicare
USPSTF: U.S. Preventive Services Task Force
